# Notes and News

**Published:** 1903-04-11

**Authors:** 


### Notes and News.

The King will open the new fever hospital at Colinton Mains during his visit to Scotland.

Their Royal Highnesses the Prince and Princess of Wales (Grand President and Lady Grand President of the League of Mercy) will receive the presidents, lady presidents, and certain others connected with the League at Marlborough House on the afternoon of Friday, May 22nd.

The Duke of Northumberland will preside at the annual meeting of the Invalid Children's Aid Association to be held at 3 Grosvenor Place on Wednesday, 29th inst., at 3 o'clock.

We are so constantly hearing of the disadvantages attaching to the Army Medical Service that it is a relief to hear recommendations of the sister service from a retired naval medical officer. In a letter to the *Times* Fleet Surgeon Gibbs says: "I think, if the many great and varied advantages of the Naval Medical Service were better known, there would be no lack of candidates for commissions in the service. And its advantages are many; the pay is good, the expenses are very small, the life is healthy. There are very great social advantages. Many parts of the world are seen under the best circumstances. The opportunities for studying the various diseases that occur in many parts of the world are very great, especially in the civil hospitals abroad, where the naval medical officer is always a welcome visitor; and last, but not least in the eyes of a medical man, the professional advantages are not only good, but often much above the average. A great deal of very good surgical work is available and is done in the many naval hospitals at home and abroad. Opportunities are given to keep up to the times by periods of study at the civil hospitals; all the apparatus provided both afloat and ashore is of the latest type and fully up to date, and under the present Board of Admiralty, at the instigation of the present Director-General, Sir H. Norbury, the greatest encouragement is given to excel in professional work, by specially promoting medical officers for zeal and success in their professional work."

Small-pox shows a decided increase in Dublin. The number under treatment exceeds 70.

Miss Helen Gordon Upward has bequeathed £100 each to the Royal London Ophthalmic Hospital and the Royal Hospital for Diseases of the Chest.

The late Colonel J. Gore Campbell has bequeathed £800 to the Cancer Hospital, Brompton; £600 to the Brompton Consumption Hospital; £500 to the Clapham Home for the Dying; £100 to the Home for Incurable Children, Maida Vale; and £100 to the British Home for Incurables.

The Committee on Wage-earning Children have prepared a memorandum on the Employment of Children Bill of 1903. In this memorandum they recommend that local authorities should be directed rather than given power to make bye-laws for the regulation of the employment of children.

The offer of £400,000 for the site of the Manchester Infirmary by the City Corporation has been at last accepted by the trustees of the Infirmary. The Infirmary is to be rebuilt on the much discussed Stanley Grove site, distant two miles from its present position. This site is in part a gift from the authorities of Owens College, and an important element in the removal scheme is the close connection rendered possible now with the Medical School at Owens College. An emergency receiving establishment will be retained in connection with the new Infirmary, in the centre of the city.

Mr. J. Craggs, M.V.O., has written an article in the *Times* which he intends to serve as a warning and to stimulate laggard or niggardly business firms and other corporate bodies to give more freely to the hospitals. His article is devoted to showing that should voluntary support fail, the hospitals forced on the rates would cost the ratepayers far more than they do at present. Mr. Craggs, from lack of statistics in respect to the Poor Law infirmaries, bases his arguments on the expenditure of the Hospital Asylums Board. The *Times* in a leader on Mr. Craggs' article holds that no comparison can properly be drawn between the voluntary expenditure system of general hospitals and the Institutions for Epidemics supported by the Asylums Board; and this because the fever hospitals must maintain, in times of slackness, the staff necessary to deal with an epidemic. There is certainly ground for this defence, and had Mr. Craggs commented upon the expenditure on buildings and contracts rather than upon salaries and upkeep, his argument might not have been challenged. As it is, we feel that in regard especially to the expenditure on small-pox the anti-vaccinators and conscientious objectors are responsible rather than the Metropolitan Asylums Board. Indeed we regard it as a misfortune to the voluntary cause which Mr. Craggs advocates that he has not been able to draw his lessons from the Poor Law system instead of that of the Metropolitan Asylums Board. However, he promises a fuller treatise in pamphlet form, and from this we hope to glean further points and more information than he has been able to give us in his able article in the columns of the *Times*.

